# Demography, baseline disease characteristics, and treatment history of psoriasis patients with self-reported psoriatic arthritis enrolled in the PSOLAR registry

**DOI:** 10.1186/s41927-018-0034-7

**Published:** 2018-09-29

**Authors:** Arthur Kavanaugh, Kim Papp, Alice B. Gottlieb, Elke M. G. J. de Jong, Soumya D. Chakravarty, Shelly Kafka, Wayne Langholff, Kamyar Farahi, Bhaskar Srivastava, Jose U. Scher

**Affiliations:** 1 Division of Rheumatology, Allergy and Immunology, Department of Medicine, University of California, San Diego, CA USA; 2 K Papp Clinical Research and Probity Medical Research Inc., Waterloo, ON Canada; 3 New York Medical College, Metropolitan Hospital, New York, NY USA; 4 Department of Dermatology, Radboud University Medical Center, and Radboud University, Nijmegen, The Netherlands; 5 Janssen Scientific Affairs, LLC, Horsham, PA USA; 6 Drexel University College of Medicine, Philadelphia, PA USA; 7 Janssen Research & Development, LLC, Spring House, PA USA; 8 Division of Rheumatology, New York University School of Medicine and Hospital for Joint Diseases, 301 East 17th Street, Room 1608, New York, NY 10003 USA

**Keywords:** Demography, PSOLAR, Psoriasis, Psoriatic arthritis

## Abstract

**Background:**

To evaluate demographics, family history, and previous medication use at enrollment in a subset of psoriasis patients with self-reported psoriatic arthritis (PsA) enrolled in Psoriasis Longitudinal Assessment and Registry (PSOLAR).

**Methods:**

PSOLAR is an international, prospective, longitudinal, disease-based registry that collects data in patients receiving, or are eligible to receive, systemic or biologic treatments for psoriasis. Baseline demographic, disease characteristics, medical history, and prior medication use at enrollment were evaluated in PSOLAR psoriasis patients self-reporting PsA (*n* = 4315); a subset of which had their diagnosis of PsA established by a healthcare provider (HCP; *n* = 1719); patients with psoriasis only (*n* = 7775); and the overall PSOLAR population (*n* = 12,090).

**Results:**

At enrollment, demographic characteristics were distinct between psoriasis patients self-reporting PsA and psoriasis only patients. Of the patients with psoriasis self-reporting PsA, 44.4% had cardiovascular disease (CVD), 26.3% had psychiatric illness, and 3.2% had inflammatory bowel disease (IBD), with each more prevalent than among patients with psoriasis only (*p* < 0.001). Overall, 17.5% of psoriasis patients self-reporting PsA had a family history of PsA, 29.8% had used systemic steroids, 39.5% had used nonsteroidal anti-inflammatory drugs, and 83.5% had used biologics.

**Conclusions:**

Demographics, family history, and previous medication use were generally comparable between “PsA established by a HCP” patients and psoriasis patients self-reporting PsA in the PSOLAR registry, but there were statistical differences compared with the psoriasis only group regarding the prevalence of certain comorbidities (CVD, psychiatric illness, and IBD). These analyses provide important data regarding characteristics of psoriasis patients with self-reported PsA in PSOLAR.

**Trial registration:**

NCT00508547.

**Electronic supplementary material:**

The online version of this article (10.1186/s41927-018-0034-7) contains supplementary material, which is available to authorized users.

## Background

Psoriatic arthritis (PsA) is a chronic immune-mediated inflammatory arthritis often characterized by inflammation of the peripheral joints, nail changes, enthesitis, and dactylitis, and is classified with the spondyloarthropathies due to the presence of spondylitis in up to 40% of patients [1]. Psoriasis is commonly associated with PsA, with up to 30% of patients with psoriasis developing PsA [2, 3]. Psoriasis usually precedes the onset of PsA by 7 to 12 years in approximately 75–84% of patients who develop PsA [4]. Because most patients develop psoriasis first, they are often treated by a dermatologist, general practitioner, or other health care provider who may not have extensive experience in evaluating patients for symptoms of PsA [3, 5]. Commonly, the treating physician refers the patient to a rheumatologist to make a PsA diagnosis only after the patient reports symptomatic musculoskeletal involvement. This delay in diagnosis and lack of recognition of PsA symptoms accounts for many cases of undiagnosed PsA in this patient population [5]. In fact, a recent study suggests that approximately 15% of patients with psoriasis have undiagnosed PsA [4]. In addition to psoriasis, other comorbidities (e.g., obesity, cardiovascular risk, diabetes, hypertension, and gastrointestinal disorders) are associated with PsA [6–9]. Beyond the physical effects, the psychological well being and health-related quality of life (HRQoL) of PsA patients are often negatively impacted [10–12].

Due to the complexity and underdiagnosis of PsA, it is important to understand the typical profile of patients with PsA by considering demographics, disease characteristics, medical and family history, lifestyle risk factors, and prior treatment use. Observational studies allow for collection of data in a real-world setting [13, 14]. The Psoriasis Longitudinal Assessment and Registry (PSOLAR) is a large, multicenter, international, longitudinal, disease-based registry with prospective enrollment of approximately 12,000 patients with psoriasis who are receiving, or are candidates for treatment with, systemic therapies for psoriasis [15, 16]. Among a limited number of registries containing data for patients with PsA [17–19], PSOLAR is a mature, well-validated registry that allows for the collection of data prospectively from psoriasis patients with self-reported PsA.

Here, we report baseline demographics, psoriasis disease characteristics, medical history, and prior medication use among patients with psoriasis and self-reported PsA enrolled in PSOLAR. These analyses provide information for characterizing self-reported PsA patients and help provide context for reporting longitudinal safety data collected from these patients in the registry.

## Methods

### Patients and study design

The details of the design of PSOLAR have been previously reported [15]. Overall, adult patients (≥18 years) were eligible if they had a diagnosis of psoriasis for which they were candidates for or currently receiving treatment with a systemic agent; patients could also have any overlapping forms of psoriasis, including PsA [15]. As of August 23, 2015, PSOLAR was fully enrolled with 12,090 patients, totaling 48,870 patient-years of follow-up.

In PSOLAR, demographic and psoriasis disease characteristics, medical, social, and family histories, and previous medication use were collected at each site using electronic case report forms. Data were collected (mainly at site visits) at baseline and every-6-months, except medical, social, and family histories, which were only collected at baseline. Due to the original design of the study, only psoriasis disease activity measures were collected and not those for PsA. Patients were also asked for additional data for some responses, including whether patients self-reported having PsA, and, additionally, if their healthcare provider (HCP; e.g., rheumatologist, dermatologist, etc.) established a diagnosis of PsA, but with no further confirmation by the investigator. From hereon in this report, psoriasis patients that self-reported both their PsA and its evaluation by a HCP (“PsA established by a HCP”) will be defined as such.

### Statistical analysis

All patients from the PSOLAR registry at the time of this analysis (August 23, 2015) were separated into three groups: 1) patients self-reporting PsA, which was established by a HCP, 2) patients self-reporting PsA, and 3) patients with psoriasis only. Baseline demographics and disease characteristics at enrollment were summarized for each group. The Pearson chi square test was used to detect significant differences between the two groups for categorical variables. Although all *p*-values reported here are unadjusted, and not specifically controlled for multiplicity, only comparisons with a *p*-value of < 0.001 were considered statistically significant. A Bonferroni adjusted *p*-value controlling for multiplicity would be approximately 0.0022. In addition, only clinically meaningful differences were identified in the manuscript.

## Results

### Baseline demographics and disease characteristics

**Fig. 1:**
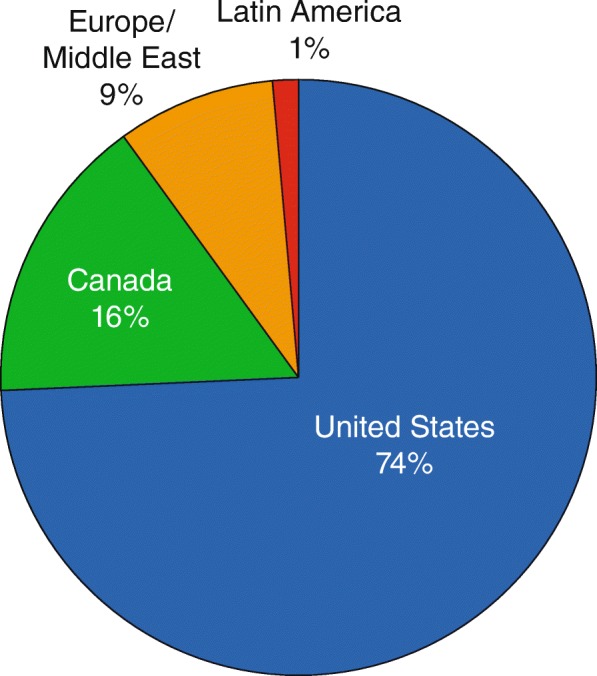
Proportion of all patients enrolled in PSOLAR by geographic region

A total of 12,090 patients with psoriasis were enrolled in PSOLAR at the time of this analysis, (August 23, 2015) of which 4315 psoriasis patients self-reported having PsA, accounting for approximately 36% of the total PSOLAR registry population. Of these 4315 psoriasis patients who self-reported having PsA, a smaller subset of 1719 patients self-reported that their HCP established a diagnosis of PsA. Overall, the demographic and clinical characteristics of this smaller subset of patients were similar to those for the overall population with self-reported PsA group (Table 1). The treatment groups included in this analysis were defined as: psoriasis patients self-reporting PsA, established by a HCP (*n* = 1719); psoriasis patients self-reporting PsA (*n* = 4315); patients with psoriasis only (*n* = 7775); and the overall PSOLAR population (all evaluable psoriasis patients with and without PsA, *n* = 12,090). Most PSOLAR patients (74%) were enrolled in sites in the United States, with 16% in Canada, 9% in the European Union/Middle East, and 1% in Latin America (Fig. 1).

**Table 1:** Baseline demographics and disease characteristics; PSOLAR psoriasis patients

	Psoriasis patients self-reporting PsA, established by a HCP^a^	Psoriasis patients self-reporting PsA^b^	Patients with psoriasis only^c^	All PSOLAR patients^d^
Patients enrolled, N	1719	4315	7775	12,090
Years since psoriasis diagnosis				
N	1707	4287	7692	11,979
Mean ± SD	21.1 ± 13.3	19.7 ± 13.5	16.3 ± 13.3	17.5 ± 13.4
Median	19.8	18.3	13.7	15.2
Age, years				
N	1719	4315	7774	12,089
Mean ± SD	50.8 ± 12.1	50.4 ± 12.6	47.6 ± 14.5	48.6 ± 13.9
Median	51.0	51.0	48.0	49.0
Sex, N	1719	4315	7775	12,090
Men	894 (52.0)	2194 (50.8)	4441 (57.1)	6635 (54.9)
Race, N	1719	4315	7774	12,089
White	1484 (86.3)	3622 (83.9)	6401 (82.3)	10,023 (82.9)
Weight, kg				
N	1682	4255	7663	11,918
Mean ± SD	92.7 ± 22.9	92.2 ± 23.1	89.2 ± 22.6	90.2 ± 22.9
Median	90.0	89.9	86.3	88.0
Body mass index (BMI), kg/m^2^				
N	1679	4248	7656	11,904
Mean ± SD	31.9 ± 7.1	31.8 ± 7.3	30.4 ± 7.1	30.9 ± 7.2
Median	30.84	30.68	29.21	29.71
Obesity class	1679	4248	7656	11,904
Underweight (BMI < 18.5)	5 (0.3)	15 (0.4)	46 (0.6)	61 (0.5)
Normal (18.5–24.9)	244 (14.5)	645 (15.2)	1631 (21.3)	2276 (19.1)
Overweight (25.0–29.9)	501 (29.8)	1296 (30.5)	2536 (33.1)	3832 (32.2)
Obesity class I (30.0–34.9)	438 (26.1)	1094 (25.8)	1808 (23.6)	2902 (24.4)
Obesity class II (35.0–39.9)	265 (15.8)	654 (15.4)	906 (11.8)	1560 (13.1)
Obesity class III (40.0 +)	226 (13.5)	544 (12.8)	729 (9.5)	1273 (10.7)

Values are presented as n (%) unless noted otherwise

*HCP* healthcare provider, *PsA* psoriatic arthritis, *SD* standard deviation

^a^A subset of PSOLAR psoriasis patients self-reporting PsA, established by a HCP

^b^PSOLAR psoriasis patients with self-reported PsA

^c^PSOLAR psoriasis patients not self-reporting PsA

^d^Includes all PSOLAR patients with psoriasis who may or may not have PsA

Overall, the demographic characteristics of PSOLAR patients at enrollment were different between all psoriasis patients self-reporting PsA and patients with psoriasis only (Table 1). Of the psoriasis patients self-reporting PsA, most were white (83.9%), 50.8% were men with a mean age of approximately 50 years. At enrollment, approximately 85% of patients self-reporting PsA were overweight or obese, with a mean weight of 92.2 kg. More patients with self-reported PsA were categorized in obesity classes II/II (35.0–40.0+ kg) than patients with psoriasis only (Table 1). Overall, PsA patients had a longer duration of psoriasis (time since diagnosis) (19.7 ± 13.5 years) than patients with psoriasis only (16.3 ± 13.3 years).

Due to the original design of the registry, disease activity for psoriasis, but not PsA, was collected at enrollment in PSOLAR. Psoriasis disease activity at entry into the registry was similar between “PsA established by a HCP” patients and psoriasis patients self-reporting PsA. The majority of psoriasis patients self-reporting PsA 96.8% had plaque psoriasis, with approximately 12.5% body surface area (BSA) involvement of the skin and mean Physicians’ Global Assessment scores of 2 (indicating mild psoriasis) (Additional file 1: Table S1).

### Medical history of special interest at enrollment

**Fig. 2:**
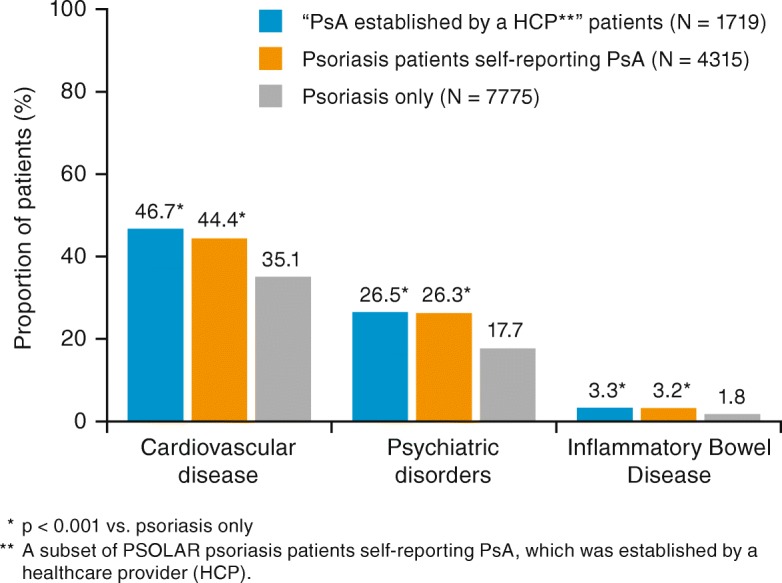
Proportion (%) of PSOLAR patients with cardiovascular disease, psychiatric disorders, or inflammatory bowel disease. PsA, psoriatic arthritis

Comorbidities occur frequently in patients with PsA and patients with psoriasis. Of particular interest are the associations of PsA with cardiovascular disease (CVD), psychiatric disorders, and inflammatory bowel disease (IBD). Overall, 46.7% of patients with “PsA established by a HCP” and 44.4% of psoriasis patients self-reporting PsA had CVD, which was significantly greater than the proportion with psoriasis only (35.1%; each *p* < 0.001) (Table 2; Fig. 2). The proportion of patients with psychiatric disorders was significantly greater in the “PsA established by a HCP” patients (26.5%) compared with patients with psoriasis only (17.7%; each *p* < 0.001) (Table 2; Fig. 2). Depression (19.0%) and anxiety (14.3%) were the most commonly reported psychiatric disorders. Similarly, more “PsA established by a HCP” patients (3.3%) and psoriasis patients with self-reported PsA (3.2%) had IBD compared with patients with psoriasis only (1.8%; each *p* < 0.001) (Table 2; Fig. 2). Differences in the prevalence of other comorbidities, smoking, and alcohol use were also reported between psoriasis patients with self-reported PsA and patients with psoriasis only (Additional file 2: Table S2).

**Table 2:** Medical history of special interest at enrollment; PSOLAR psoriasis patients

	Psoriasis patients self-reporting PsA, established by a HCP^a^ (*N* = 1719)	Psoriasis patients self-reporting PsA^b^ (*N* = 4315)	Patients with psoriasis only^c^ (*N* = 7775)	All PSOLAR patients^d^(*N* = 12,090)
Number of patients with medical history data	1719	4315	7772	12,087
Cardiovascular	802 (46.7)*	1916 (44.4)*	2728 (35.1)	4644 (38.4)
Hypertension	639 (37.2)	1522 (35.3)	2094 (26.9)	3616 (29.9)
Hyperlipidemia	429 (25.0)	955 (22.1)	1334 (17.2)	2289 (18.9)
Atherosclerotic disease	80 (4.7)	203 (4.7)	230 (3.0)	433 (3.6)
Coronary artery disease	72 (4.2)	178 (4.1)	192 (2.5)	370 (3.1)
Myocardial infarction	58 (3.4)	125 (2.9)	169 (2.2)	294 (2.4)
Angina	36 (2.1)	82 (1.9)	86 (1.1)	168 (1.4)
Transient ischemic attack/stroke	28 (1.6)	66 (1.5)	89 (1.1)	155 (1.3)
Congestive heart failure	29 (1.7)	62 (1.4)	64 (0.8)	126 (1.0)
Peripheral arterial disease	13 (0.8)	32 (0.7)	39 (0.5)	71 (0.6)
Psychiatric disorders	455 (26.5)*	1135 (26.3)*	1376 (17.7)	2511 (20.8)
Depression	342 (19.9)	822 (19.0)	968 (12.5)	1790 (14.8)
Anxiety	246 (14.3)	618 (14.3)	732 (9.4)	1350 (11.2)
Bipolar	31 (1.8)	81 (1.9)	102 (1.3)	183 (1.5)
Suicidal ideation	33 (1.9)	64 (1.5)	53 (0.7)	117 (1.0)
Schizophrenia	5 (0.3)	11 (0.3)	19 (0.2)	30 (0.2)
Inflammatory bowel disease	56 (3.3)*	138 (3.2)*	139 (1.8)	277 (2.3)
Indeterminate colitis	22 (1.3)	54 (1.3)	52 (0.7)	106 (0.9)
Ulcerative colitis	19 (1.1)	45 (1.0)	48 (0.6)	93 (0.8)
Crohn’s disease	10 (0.6)	29 (0.7)	32 (0.4)	61 (0.5)

Data are presented as n (%)

*HCP* healthcare provider, *PsA* psoriatic arthritis

*p < 0.001 vs. patients with psoriasis only

*P*-values for categorical variables were obtained from Pearson chi-square test with patients with psoriasis only as a reference

^a^A subset of PSOLAR psoriasis patients self-reporting PsA, established by a HCP

^b^PSOLAR psoriasis patients with self-reported PsA

^c^PSOLAR psoriasis patients not self-reporting PsA

^d^Includes all PSOLAR patients with psoriasis who may or may not have PsA

### Family medical history

The proportion of patients with a family history of PsA was comparable between the “PsA established by a HCP” patients (16.4%) and those self-reporting PsA (17.5%), but both were significantly greater than patients with psoriasis only (6.3%; each *p* < 0.001; Table 3). A significantly greater proportion of “PsA established by a HCP” patients (53.3%) and psoriasis patients self-reporting PsA (50.1%) had a family history of CVD compared with patients with psoriasis only (41.5%) (each *p* < 0.001; Table 3). Similarly, a significantly greater proportion of “PsA established by a HCP” patients (8.1%) and psoriasis patients in the group self-reporting PsA (8.2%) had a family history of IBD, compared with the group with psoriasis only (5.6%) (each *p* < 0.001). Overall, greater proportions of psoriasis patients with self-reported PsA had family histories of comorbidities compared with patients with psoriasis only (Table 3).

**Table 3:** Family medical history

	Psoriasis patients self-reporting PsA, established by a HCP^a^ (*N* = 1719)	Psoriasis patients self-reporting PsA^b^ (*N* = 4315)	Patients with psoriasis only^c^ (*N* = 7775)	All PSOLAR patients^d^ (*N* = 12,090)
Patients with family history data, N	1676	4205	7520	11,725
Psoriatic arthritis	275 (16.4)*	736 (17.5)*	471 (6.3)	1207 (10.3)
Cardiovascular disease	894 (53.3)*	2105 (50.1)*	3124 (41.5)	5229 (44.6)
Psoriasis	837 (49.9)*	2041 (48.5)*	3286 (43.7)	5327 (45.4)
Inflammatory bowel disease	135 (8.1)	345 (8.2)*	423 (5.6)	768 (6.6)
Indeterminate colitis	48 (2.9)	94 (2.2)	108 (1.4)	202 (1.7)
Crohn’s disease	40 (2.4)	75 (1.8)	125 (1.7)	200 (1.7)
Ulcerative colitis	41 (2.4)	62 (1.5)	94 (1.3)	156 (1.3)
Sprue/Celiac disease	7 (0.4)	12 (0.3)	30 (0.4)	42 (0.4)
Diabetes	683 (40.8)**	1755 (41.7)*	2768 (36.8)	4523 (38.6)
Other cancer	640 (38.2)*	1508 (35.9)*	2393 (31.8)	3901 (33.3)
Asthma	371 (22.1)*	918 (21.8)*	1338 (17.8)	2256 (19.2)
Thyroid disease	307 (18.3)*	771 (18.3)*	1114 (14.8)	1885 (16.1)
Non-melanoma skin cancer	203 (12.1)**	518 (12.3)*	749 (10.0)	1267 (10.8)
Rashes	152 (9.1)	304 (7.2)^ns^	464 (6.2)	768 (6.6)
Melanoma skin cancer	102 (6.1)**	268 (6.4)*	367 (4.9)	635 (5.4)
Multiple sclerosis	30 (1.8)	99 (2.4)*	112 (1.5)	211 (1.8)
Vitiligo	47 (2.8)**	103 (2.4)^ns^	128 (1.7)	231 (2.0)
None	127 (7.6)	369 (8.8)*	992 (13.2)	1361 (11.6)

Values are n (%)

*HCP* healthcare provider, *PsA* psoriatic arthritis, *ns* not significant

*p < 0.001 vs. patients with psoriasis only

** *p* < 0.05 vs. patients with psoriasis only

^ns^ indicates non-significant for psoriasis patients self-reporting PsA vs. patients with psoriasis only

*P*-values for categorical variables were obtained from Pearson chi-square test with patients with psoriasis only as a reference

^a^A subset of PSOLAR psoriasis patients self-reporting PsA, established by a HCP

^b^PSOLAR psoriasis patients with self-reported PsA

^c^PSOLAR psoriasis patients not self-reporting PsA

^d^Includes all PSOLAR patients with psoriasis who may or may not have PsA

### Previous medication use

At enrollment, approximately 30% of psoriasis patients self-reporting PsA had used systemic steroids, which was significantly greater than the proportion of patients with psoriasis only (19.9%; *p* < 0.001) (Table 4). The proportion of “PsA established by a HCP” patients (48.6%) and psoriasis patients self-reporting PsA (39.5%) that had used nonsteroidal anti-inflammatory drugs (NSAIDs), was also significantly higher than in patients with psoriasis only (7.0%; each *p* < 0.001). Immunomodulators had been used by a significantly greater proportion of “PsA established by a HCP” patients (69.6%) and psoriasis patients self-reporting PsA (59.6%), compared with the psoriasis only population (each *p* < 0.001; 41.5%). As expected, methotrexate and cyclosporine use was greater among psoriasis patients with self-reported PsA than among patients with psoriasis only. Approximately 85% of psoriasis patients with self-reported PsA had used biologics, which was significantly greater than the proportion of patients with psoriasis only (66.4%; *p* < 0.001). The high prevalence of biologic use among PsA patients is not unexpected, given the long duration of their psoriasis (“PsA established by a HCP” patients: 21.1 ± 13.3 years; psoriasis patients self-reporting PsA: 19.7 ± 13.5 years; psoriasis only: 16.3 ± 13.3 years; Table 1). Approximately 40% of all psoriasis patients self-reporting PsA had received one biologic medication prior to enrollment, which was comparable to patients with psoriasis only (38.5%). More than one-quarter of psoriasis patients with self-reported PsA had received two biologics, which was greater than the proportion reported for patients with psoriasis only (17.6%). Etanercept, adalimumab, and infliximab were the most commonly used biologics among patients with PsA (Table 4).

**Table 4:** Medication use at enrollment; PSOLAR psoriasis patients

	Psoriasis patients self-reporting PsA, established by a HCP^a^ (*N* = 1719)	Psoriasis patients self-reporting PsA^b^ (*N* = 4315)	Patients with psoriasis only^c^ (*N* = 7775)	All PSOLAR patients^d^ (*N* = 12,090)
Patients with data, N	1714	4306	7762	12,068
Systemic steroids	566 (33.0)*	1284 (29.8)*	1548 (19.9)	2832 (23.5)
Retinoids and/or combination topical	760 (44.3)	2001 (46.5)*	3285 (42.3)	5286 (43.8)
Taclonex	367 (21.4)	1146 (26.6)	1804 (23.2)	2950 (24.4)
Acitretin	406 (23.7)	921 (21.4)	1506 (19.4)	2427 (20.1)
Tazorac	164 (9.6)	561 (13.0)	758 (9.8)	1319 (10.9)
Etretinate	25 (1.5)	50 (1.2)	53 (0.7)	103 (0.9)
Patients with NSAID data, N	1718	4310	7769	12,079
NSAIDs	835 (48.6)*	1702 (39.5)*	540 (7.0)	2242 (18.6)
Sulfasalazine	112 (6.5)	162 (3.8)	26 (0.3)	188 (1.6)
Other NSAIDs	781 (45.5)	1626 (37.7)	520 (6.7)	2146 (17.8)
Immunomodulators	1193 (69.6)*	2567 (59.6)*	3221 (41.5)	5788 (48.0)
Methotrexate	1104 (64.4)	2326 (54.0)	2634 (33.9)	4960 (41.1)
Cyclosporine	339 (19.8)	738 (17.1)	1161 (15.0)	1899 (15.7)
Oral Tacrolimus	1 (0.1)	3 (0.1)	4 (0.1)	7 (0.1)
Mycophenolate mofetil	10 (0.6)	25 (0.6)	22 (0.3)	47 (0.4)
Other Immunomodulators	60 (3.5)	131 (3.0)	147 (1.9)	278 (2.3)
Any biologic medications	1464 (85.2)*	3603 (83.5)*	5164 (66.4)	8767 (72.5)
History of biologic medications at entry				
Etanercept	893 (51.9)	2305 (53.4)	2581 (33.2)	4886 (40.4)
Adalimumab	678 (39.4)	1673 (38.8)	1890 (24.3)	3563 (29.5)
Infliximab	477 (27.7)	1037 (24.0)	895 (11.5)	1932 (16.0)
Ustekinumab	378 (22.0)	703 (16.3)	1569 (20.2)	2272 (18.8)
Efalizumab	147 (8.6)	452 (10.5)	892 (11.5)	1344 (11.1)
Alefacept	96 (5.6)	272 (6.3)	428 (5.5)	700 (5.8)
Golimumab	32 (1.9)	44 (1.0)	2 (0.0)	46 (0.4)
Other	48 (2.8)	87 (2.0)	147 (1.9)	234 (1.9)
Number of biologic medications used prior to entry				
0	255 (14.8)*	712 (16.5)*	2611 (33.6)	3323 (27.5)
1	682 (39.7)	1734 (40.2)	2992 (38.5)	4726 (39.1)
2	445 (25.9)	1097 (25.4)	1366 (17.6)	2463 (20.4)
3	222 (12.9)	525 (12.2)	592 (7.6)	1117 (9.2)
≥ 4	115 (6.7)	247 (5.7)	214 (2.8)	461 (3.8)
Topical therapy	1668 (97.3)	4207 (97.7)*	7485 (96.4)	11,692 (96.9)
Topical steroid therapy	1637 (95.5)	4142 (96.2)	7325 (94.4)	11,467 (95.0)
High potency	1373 (80.1)	3607 (83.8)	6156 (79.3)	9763 (80.9)
Medium potency	1049 (61.2)	2680 (62.2)	4481 (57.7)	7161 (59.3)
Low potency	707 (41.2)	1826 (42.4)	2749 (35.4)	4575 (37.9)
Phototherapy	1028 (60.0)*	2411 (56.0)^ns^	4172 (53.7)	6583 (54.5)
Psoralens + UVA	417 (24.3)	860 (20.0)	1207 (15.6)	2067 (17.1)
UVB	833 (48.6)	2000 (46.4)	3524 (45.4)	5524 (45.8)
Laser	29 (1.7)	56 (1.3)	129 (1.7)	185 (1.5)

Values are n (%)

*HCP* healthcare provider, *NSAID* nonsteroidal anti-inflammatory drug, *ns* not significant, *PsA* psoriatic arthritis, *UVA/UVB* ultraviolet A/B

*p < 0.001 vs. patients with psoriasis only

^ns^ indicates non-significant for psoriasis patients self-reporting PsA vs. patients with psoriasis only

*P*-values for categorical variables were obtained from Pearson chi-square test with patients with psoriasis only as a reference

^a^A subset of PSOLAR psoriasis patients self-reporting PsA, established by a HCP

^b^PSOLAR psoriasis patients with self-reported PsA

^c^PSOLAR psoriasis patients not self-reporting PsA

^d^Includes all PSOLAR patients with psoriasis who may or may not have PsA

## Discussion

Registries provide real-world information beyond what is captured in a randomized clinical trial setting [20, 21]. A total of 12,090 patients with psoriasis (with 48,870 patient-years of follow-up) have been enrolled in the PSOLAR registry [15] as of August 2015, making it the largest psoriasis registry to date. It has been reported that up to 30% of patients with psoriasis also develop PsA [2, 17], which was consistent with the proportion of psoriasis patients self-reporting PsA in PSOLAR. Given the prevalence of PsA among patients with psoriasis, it is important for HCPs treating psoriasis patients to understand the phenotypic profile of these patients with self-reported PsA in a real-world setting.

Patients with psoriasis and PsA often have comorbidities (CVD, psychiatric disease, IBD) and deleterious social behaviors (smoking/alcohol consumption) that can impact treatment decisions [22] and HRQoL [23, 24]. However, observations from PSOLAR indicate that a greater proportion of psoriasis patients self-reporting PsA had CVD compared with those with only psoriasis. Our findings are consistent with other studies reporting that significant cardiovascular comorbidities are associated with PsA [25, 26], and that the prevalence of CVD is significantly greater in patients with PsA compared with the general population [27, 28]. Also, a higher proportion of psoriasis patients self-reporting PsA described psychiatric disorders, including depression and anxiety, compared with patients with psoriasis only. This supports findings from other studies in which the rates of anxiety and depression were significantly higher in patients with PsA versus those without PsA [29] and even when compared with patients with rheumatoid arthritis [12]. Furthermore, a greater proportion of psoriasis patients with self-reported PsA than with psoriasis only indicated having IBD. This is especially important because patients with PsA and IBD may be at serious risk for developing other comorbidities [7, 30–32]. Our findings from PSOLAR support the prevalence of IBD in psoriasis patients with PsA that has been reported in other studies [33, 34], thereby adding to the validity and confidence of conducting PsA-centric analyses in the context of larger psoriasis registries.

As expected, the proportion of patients who had used NSAIDs was highest among the groups of psoriasis patients with self-reported PsA, which was considerably higher than patients with psoriasis only. Similarly, the use of systemic steroids and synthetic immunomodulators (especially methotrexate) was much higher among psoriasis patients with self-reported PsA. A greater proportion of psoriasis patients with self-reported PsA used biologics, particularly tumor necrosis factor inhibitors, compared with patients with psoriasis only. This finding may further validate that these patients may actually have PsA, but could also reflect potential treatment selection bias inherent to a registry, and may not be reflective of typical rheumatology clinical practice. As expected, retinoids and/or combination topical therapies were more commonly used by patients with psoriasis only, as these treatments are not effective for PsA.

Despite differences observed between patients with self-reported PsA and patients with psoriasis in PSOLAR, the data from the two subsets of patients with self-reported PsA (i.e., “PsA established by a HCP” and self-reported) were similar to each other. Some potential limitations of the “PsA established by a HCP” group include: 1) this group had a smaller number of patients and 2) these patients self-reported having a HCP establish their diagnosis of PsA (which could have included, but was not limited to, a rheumatologist or a dermatologist). These baseline disease characteristics are more reflective of an active psoriasis population, on which the PSOLAR registry is based. Overall, baseline demographics and psoriasis disease characteristics were comparable between the two groups; exceptions included a slightly higher proportion of “PsA established by a HCP” patients having a family history of cancer versus patients self-reporting PsA, and a greater number of “PsA established by a HCP” patients having higher NSAIDs use than patients self-reporting PsA.

There are several limitations to consider when interpreting data from this analysis of the PSOLAR registry. As with any retrospective analysis, reporting and recall biases may exist, including exposure to treatment at baseline, which may reveal prior treatment selection bias. The increased use of biologics is likely to be a result of the enrollment of patients with more active/severe psoriasis (i.e., high BSA involvement), who are deemed appropriate candidates for biologic treatment earlier in their disease course. PSOLAR longitudinally follows advanced, active psoriasis patients, and the subset of psoriasis patients with self-reported PsA in this study may not necessarily reflect those typically treated by the rheumatology community at large. Of note, there was a target goal for enrolling patients receiving ustekinumab or infliximab, which led to enhanced enrollment of patients receiving biologics compared with the general psoriasis population. Additionally, 90% of the patients enrolled in PSOLAR were from North America (United States and Canada); however, the large number of patients enrolled (12,090) allowed for a wide representation of patients with varying disease characteristics and medical histories.

Similarly, PsA disease activity measures at baseline and longitudinally were not collected, but rather, only those for psoriasis disease activity. However, of note, recently published registry data that focused on characterizing the relationship between skin severity (as defined by BSA) and joint activity (as defined by Clinical Disease Activity Index) in patients with both psoriasis and PsA at enrollment suggested a potential correlation between the two [35]. Nevertheless, in PSOLAR, no data were collected to determine if psoriasis patients self-reporting PsA met specific disease classification criteria (e.g., Classification Criteria for Psoriatic Arthritis) nor disease activity measures. Similarly, as originally designed as a psoriasis registry, PSOLAR does not capture the evolution of patients with psoriasis who may go on to develop PsA, which represents a particularly relevant knowledge gap in the field. Future databases and related efforts (particularly if containing biosamples) should include tools to prospectively diagnose PsA in psoriasis patient cohorts to attempt to elucidate clinical, demographic, and mechanistic determinants of progression in the psoriatic disease continuum.

## Conclusions

We assessed baseline demographics, disease characteristics, medical, social, and family medical histories, as well as previous treatment use in psoriasis patients with self-reported PsA in PSOLAR, a large psoriasis registry. This analysis confirms that psoriasis patients with PsA often have serious comorbidities, some of which, such as CVD, psychiatric disorders, and IBD, were statistically more prevalent in psoriasis patients with self-reported PsA than patients with psoriasis only. Despite several differences noted between patients with psoriasis and patients with self-reported PsA in PSOLAR, no notable differences were observed between the subset of “PsA established by a HCP” patients and those with self-reported PsA, providing a rationale for the use of self-reported PsA cohort data for future long-term safety analyses. This analysis provides additional information that will be useful for health care providers to better understand patients with PsA in a real-world setting.

## Additional files

Additional file 1 **Table S1.** Psoriasis disease activity at enrollment; PSOLAR psoriasis patients. (DOCX 26 kb)

Additional file 2 **Table S2.** Other medical and social history of psoriasis patients enrolled in PSOLAR. (DOCX 28 kb)

## Abbreviations

BSA: body surface area
CVD: cardiovascular disease
HCP: healthcare provider
HRQoL: health-related quality of life
IBD: inflammatory bowel disease
NSAIDs: nonsteroidal anti-inflammatory drugs
PsA: psoriatic arthritis
PSOLAR: Psoriasis Longitudinal Assessment and Registry
