# Supplementary material for: Demography, baseline disease characteristics, and treatment history of psoriasis patients with self-reported psoriatic arthritis enrolled in the PSOLAR registry
Source: BMC Rheumatol. 2018 Sep 29;2:29. doi: 10.1186/s41927-018-0034-7 (PMC6390609; doi:10.1186/s41927-018-0034-7)
Supplement: Supplementary file 1 — Table S1. Psoriasis disease activity at enrollment; PSOLAR psoriasis patients. (DOCX 26 kb) [file 41927_2018_34_MOESM1_ESM.docx]

**Table S1.** **Psoriasis disease activity at enrollment; PSOLAR psoriasis patients**

|  | Psoriasis patients self-reporting PsA^1^  (N=4315) | Patients with psoriasis only^2^  (N=7775) | All PSOLAR patients^3^  (N=12090) |
| --- | --- | --- | --- |
| Number of patients with data | 4311 | 7770 | 12081 |
|  |  |  |  |
| Psoriasis Type |  |  |  |
| Plaque | 4194 (97.3) | 7505 (96.6) | 11699 (96.8) |
| Other | 372 (8.6) | 747 (9.6) | 1119 (9.3) |
|  |  |  |  |
| BSA by palm method (% involvement) |  |  |  |
| N | 4273 | 7704 | 11977 |
| Mean ± SD | 12.5 ± 18.8 | 11.8 ± 16.8 | 12.1 ± 17.6 |
| Median | 5.0 | 5.0 | 5.0 |
|  |  |  |  |
| PGA Score |  |  |  |
| N | 4153 | 7415 | 11568 |
| Mean ± SD | 2.0 ± 1.2 | 2.0 ± 1.2 | 2.0 ± 1.2 |
| Median | 2.0 | 2.0 | 2.0 |
|  |  |  |  |
| PGA score | 4153 | 7415 | 11568 |
| 0 - clear | 477 (11.5) | 907 (12.2) | 1384 (12.0) |
| 1 - minimal | 940 (22.6) | 1736 (23.4) | 2676 (23.1) |
| 2 - mild | 1148 (27.6) | 2096 (28.3) | 3244 (28.0) |
| 3 - moderate | 1178 (28.4) | 1998 (26.9) | 3176 (27.5) |
| 4 - marked | 329 (7.9) | 561 (7.6) | 890 (7.7) |
| 5 - severe | 81 (2.0) | 117 (1.6) | 198 (1.7) |
| Values are n (%) unless otherwise indicated.  BSA, body surface area; PsA, psoriatic arthritis; PGA, Physician’s Global Assessment; SD, standard deviation  ^1^PSOLAR psoriasis patients with self-reported PsA  ^2^PSOLAR psoriasis patients not self-reporting PsA  ^3^Includes all PSOLAR patients with psoriasis who may or may not have PsA | | | |
