# Supplementary material for: Demography, baseline disease characteristics, and treatment history of psoriasis patients with self-reported psoriatic arthritis enrolled in the PSOLAR registry
Source: BMC Rheumatol. 2018 Sep 29;2:29. doi: 10.1186/s41927-018-0034-7 (PMC6390609; doi:10.1186/s41927-018-0034-7)
Supplement: Supplementary file 2 — Table S2. Other medical and social history of psoriasis patients enrolled in PSOLAR. (DOCX 28 kb) [file 41927_2018_34_MOESM2_ESM.docx]

**Table S2.** **Other medical and social history of psoriasis patients enrolled in PSOLAR**

|  | Psoriasis patients self-reporting PsA^1^  (N=4315) | Patients with psoriasis only^2^  (N=7775) | All PSOLAR patients^3^  (N=12090) |
| --- | --- | --- | --- |
|  |  |  |  |
| Number of patients with medical history data | 4315 | 7772 | 12087 |
|  |  |  |  |
| Pulmonary | 757 (17.5) | 988 (12.7) | 1745 (14.4) |
| Asthma | 407 (9.4) | 566 (7.3) | 973 (8.0) |
| Sleep apnea | 334 (7.7) | 362 (4.7) | 696 (5.8) |
| Chronic obstructive pulmonary disease | 105 (2.4) | 141 (1.8) | 246 (2.0) |
| Pneumonitis | 21 (0.5) | 33 (0.4) | 54 (0.4) |
| Hepatic | 235 (5.4) | 273 (3.5) | 508 (4.2) |
| Liver biopsy | 117 (2.7) | 111 (1.4) | 228 (1.9) |
| Hepatitis C | 51 (1.2) | 81 (1.0) | 132 (1.1) |
| Drug induced (psoriasis treatment medication /other) | 40 (0.9) | 35 (0.5) | 75 (0.6) |
| Cirrhosis | 46 (1.1) | 52 (0.7) | 98 (0.8) |
| Hepatitis B | 29 (0.7) | 38 (0.5) | 67 (0.6) |
| Idiopathic/autoimmune | 24 (0.6) | 13 (0.2) | 37 (0.3) |
| Alcoholic | 13 (0.3) | 18 (0.2) | 31 (0.3) |
| Skin cancer | 304 (7.0) | 261 (3.4) | 745 (6.2) |
| Basal cell carcinoma | 190 (4.4) | 441 (5.7) | 451 (3.7) |
| Squamous cell carcinoma | 114 (2.6) | 179 (2.3) | 293 (2.4) |
| Melanoma | 43 (1.0) | 63 (0.8) | 106 (0.9) |
| Unknown skin cancer | 16 (0.4) | 19 (0.2) | 35 (0.3) |
| Other types of cancer | 173 (4.0) | 282 (3.6) | 455 (3.8) |
| Endocrine | 957 (22.2) | 1328 (17.1) | 2285 (18.9) |
| Diabetes mellitus type II | 580 (13.4) | 808 (10.4) | 1388 (11.5) |
| Thyroid dysfunction | 391 (9.1) | 537 (6.9) | 928 (7.7) |
| Diabetes mellitus type I | 66 (1.5) | 87 (1.1) | 153 (1.3) |
| Other disease | 1469 (34.0) | 1810 (23.3) | 3279 (27.1) |
| Drug allergy | 1150 (26.7) | 1353 (17.4) | 2503 (20.7) |
| Environmental allergy | 402 (9.3) | 559 (7.2) | 961 (8.0) |
| Lupus | 17 (0.4) | 17 (0.2) | 34 (0.3) |
| Demyelinating disease | 11 (0.3) | 12 (0.2) | 23 (0.2) |
| Multiple sclerosis | 9 (0.2) | 9 (0.1) | 18 (0.1) |
| Optic neuritis | 2 (< 0.1) | 3 (< 0.1) | 5 (< 0.1) |
| Bone marrow suppression | 5 (0.1) | 7 (0.1) | 12 (0.1) |
|  |  |  |  |
| Social activity |  |  |  |
| Alcohol | 4310 | 7763 | 12073 |
| Current use | 2642 (61.3) | 5157 (66.4) | 7799 (64.6) |
| Have used and stopped | 683 (15.8) | 1044 (13.4) | 1727 (14.3) |
| Never used | 985 (22.9) | 1562 (20.1) | 2547 (21.1) |
| Smoking | 4313 | 7770 |  |
| Current smoker | 966 (22.4) | 1917 (24.7) | 2883 (23.9) |
| Prior smoker and stopped | 1492 (34.6) | 2460 (31.7) | 3952 (32.7) |
| Never smoked | 1855 (43.0) | 3393 (43.7) | 5248 (43.4) |
| Data are presented as n (%) unless indicated otherwise.  PsA, psoriatic arthritis  ^1^PSOLAR psoriasis patients with self-reported PsA  ^2^PSOLAR psoriasis patients not self-reporting PsA  ^3^Includes all PSOLAR patients with psoriasis who may or may not have PsA | | | |
